# Adherence to Lifestyle Interventions for Treatment of Adults with Depression: A Systematic Review and Meta-Analysis

**DOI:** 10.3390/ijerph182413268

**Published:** 2021-12-16

**Authors:** Adoración Castro, Miquel Roca, Ignacio Ricci-Cabello, Mauro García-Toro, Pau Riera-Serra, Victoria Coronado-Simsic, María Ángeles Pérez-Ara, Margalida Gili

**Affiliations:** 1 Health Research Institute of the Balearic Islands (IdiSBa), Hospital Universitario Son Espases, Edificio S, 07120 Palma de Mallorca, Spain; mroca@uib.es (M.R.); ignacio.ricci@ssib.es (I.R.-C.); mauro.garcia@uib.es (M.G.-T.); pau.riera@uib.es (P.R.-S.); marian@aliaspsicologia.com (M.Á.P.-A.); mgili@uib.es (M.G.); 2 Research Institute of Health Sciences (IUNICS), University of Balearic Islands, 07122 Palma de Mallorca, Spain; v.coronado@uib.es; 3 Primary Care Prevention and Health Promotion Research Network (RedIAPP), 28029 Madrid, Spain; 4 Primary Care Research Unit of Mallorca, Balearic Islands Health Services, 07002 Palma, Spain; 5 CIBER de Epidemiología y Salud Pública (CIBERESP), 28029 Madrid, Spain

**Keywords:** lifestyle, interventions, depression, review, meta-analysis

## Abstract

The aim of this systematic review was to determine the adherence to lifestyle interventions for adults with depression and to estimate the dropout rates in trials examining the impact of these interventions. A bibliographic search was conducted in PubMed, Embase, PsycINFO, the Cochrane library, and several sources of grey literature. We included randomised controlled trials examining the impact of multiple lifestyle interventions on depressive symptomatology in adults when compared to control or other active treatments. Two reviewers independently screened citations, extracted the relevant data, and assessed the risk of bias using Cochrane tools. A random effects meta-analysis of proportions was used to summarise the proportion of participants who completed the intervention and to determine the proportion of dropouts at post-treatment assessment. Multiple subgroup analyses were also carried out. We identified six trials. The meta-analysis of proportions showed that 53% (95%CI 49% to 58%) of the participants assigned to the intervention group fully adhered to the intervention program. The weighted mean proportion of completed intervention sessions was 66%. The pooled trial dropout rate was 22% (95%CI 20% to 24%). Around half of adults with depression adhere to lifestyle interventions. Future research is needed to develop interventions to support adherence to lifestyle interventions in depressive patients.

## 1. Introduction

Depression is considered the most common mental disorder in general population [1,2], affecting more than 264 million people worldwide [3].

Regarding its comorbidity with other diseases, there is a strong association between depression and specific physical illnesses [4], with a prevalence of coexisting chronic physical illness and depressive disorders ranging from 9.3% to 23.0% [5]. Major depressive disorder is more common in patients with neurological, oncological, or liver disease [6]. These comorbidities are associated with poorer quality of life, higher mortality rates, higher economic costs, and greater disability and functional impact on the patient compared to depression or physical illness when presented alone [5,7].

Previous research has demonstrated the association between depression and lifestyle behaviours, such as sedentary lifestyle, unhealthy diet, insomnia, social withdrawal, and stress, among others [8,9,10]. For these reasons, although psychopharmacotherapy and psychotherapy are effective treatments for depression [11,12,13], it is important to contemplate “Lifestyle Medicine” techniques for the prevention and treatment of depression [14].

In fact, previous research shows that specific single lifestyle interventions based on diet, physical activity, or sleep hygiene are beneficial in patients with depression and/or depressive symptomatology [15,16,17]. Furthermore, the possible synergistic actions of these single lifestyle interventions have also been suggested to depressive patients when proposed in combination, resulting in a multicomponent intervention or multiple lifestyle interventions for depression [10,14].

In recent years, there has been an increasing interest in the study of the multiple lifestyle interventions on depressive symptomatology, and the results are promising [18,19,20]. A recent meta-analysis suggested that universal multiple-risk lifestyle interventions may produce a modest but statistically significant effect decreasing depressive symptomatology in adults, although the available evidence base is still low [18]. These findings are supported by another recent meta-analysis, which concluded that multicomponent lifestyle medicine interventions may be effective reducing depressive symptomatology at short-term follow-up [19].

Despite the promising results, little attention has been paid to the adherence to lifestyle interventions for the treatment of depression. Only one systematic review about online lifestyle interventions for the depressed population was limited to a narrative synthesis, summarising engagement measures used in the studies included [21]. However, no meta-analysis of adherence data was carried out before. Adherence to a psychological treatment is considered an important measure of its acceptability, appropriateness, and effect [22], and therefore, more research in this area is needed.

The aim of this systematic review and meta-analysis was to study the adherence to lifestyle interventions for adults with depression. Secondary aims were to explore intervention and patient characteristics associated with adherence and to estimate the dropout rates in trials of lifestyle interventions for depression in adults.

## 2. Materials and Methods

This systematic review was previously registered in the International Prospective Register of Systematic Reviews (PROSPERO) database (CRD42020184835) on 8 May 2020. This review was conducted according to the Centre for Reviews and Dissemination guidelines on conducting systematic reviews [23] and reported using the Preferred Reporting items for Systematic Reviews and Meta-Analyses (PRISMA) guidelines [24].

### 2.1. Bibliographic Searches

We conducted structured searches in PubMed, EMBASE, PsycINFO, Web of Science (Science Citation Index), and the Cochrane Library bibliographic databases. We also searched multiple sources of grey literature (Opengrey Website, Open Access Theses and Dissertations) and trial registries (Clinicaltrials.gov).

A combination of free text and MeSH terms extracted from previous reviews and terms commonly used in relevant studies identified in a scoping search was used to develop our search strategy. Search terms covered the constructs of “depression”, “lifestyle”, “physical activity”, and “diet”. The strategy was developed in PubMed (see Table A1) and then adapted to the rest of databases. All the databases were searched from inception to 15 May 2020. The reference list of all the included studies were also screened to identify additional eligible studies (backward citation search). We use EndNote X8TM to create a bibliographical database and Rayyan [25] to screen relevant records.

### 2.2. Inclusion and Exclusion Criteria

We included studies examining the impact of multiple lifestyle interventions on depressive symptomatology in adults when compared to control or other active treatments.

In terms of participants, we included studies with adult participants (aged 18 and over) with depression and/or depressive symptoms established by a diagnosis based on a structured clinical interview following internationally recognised standards (e.g., ICD, DSM) or a validated screening measure. In terms of interventions, we included studies with a treatment arm composed by two or more lifestyle behaviours, where at least one of them was healthy diet or physical activity. Lifestyle recommendations had to be the main component of the intervention. In terms of comparator, we included studies if the comparator was a control condition (e.g., waiting-list control, treatment as usual) or an active treatment (psychological or pharmacological). In terms of outcomes, we included studies that measured the adherence to the lifestyle intervention. We included randomised controlled trials (RCTs), carried out in any setting, and published in English. We excluded letters to the editor, editorials, study protocols, and conference abstracts with no full text available.

### 2.3. Selection Procedure

All the studies retrieved in the search were assessed for initial eligibility by two independent reviewers (A.C., M.A.P.A., V.C.S., P.R.S., M.G.T., M.R., and M.G.) who examined titles and abstracts. All references were distributed among the seven reviewers so that each reviewer had to review a specified number of references. They were blind to each other’s decisions. Rayyan [25] allows each reference to be evaluated by two independent reviewers; therefore, when a reference had already been evaluated twice, it did not allow more reviews. For those studies that met this initial eligibility criteria, full text articles were sought. These full text articles were subsequently examined by two independent reviewers. Disagreements were solved by a consensus or, when needed, by involving a third reviewer. We contacted authors by email when missing data were found in papers.

### 2.4. Data Extraction and Quality Assessment

Data from the selected studies were collected using a standardised data extraction form. The extracted data included descriptive characteristics of the study, characteristics of the sample, characteristics of treatment, comparators, and outcome data. We contacted with two authors [26,27] for clarification about adherence data.

Risk of bias of the included studies was assessed using the revised Cochrane Risk of Bias tool [28]. Items were scored as follows: low risk of bias, high risk of bias, or some concerns. Two independent reviewers extracted data and assessed the risk of bias. Disagreements were solved by a consensus or, when needed, by involving a third reviewer.

### 2.5. Data Analysis and Synthesis

The main outcome was intervention adherence. Given the heterogeneity of adherence measures across the studies, we followed two different approaches to measure adherence: (1) as the proportion of participants completing 100% of the intervention (if this information was not available, we then used author’s own definition of completers), and (2) as the mean proportion of sessions completed by the participants (mean of sessions completed/total number of sessions). As a secondary outcome, we estimated the trial dropout rate, which was defined as the proportion of participants who failed to complete the research protocol/total number of participants included.

In terms of data synthesis, first, we conducted a narrative and tabulated synthesis of the findings of included studies. Then, we pooled data to summarise the proportion of participants who completed the intervention (completers) divided by total number of participants randomised to the intervention group. We used the STATA command “metaprop” [29] to pool estimates of proportions with corresponding 95% confidence intervals. Proportions were computed on the base of the Freeman–Tukey double arcsine transformation [30,31] within a random effect model framework. Heterogeneity was quantified by the I2 statistic, where I2 > 50% was deemed as substantial heterogeneity [32]. Publication bias was examined with funnel plots and the presence of asymmetry was tested with Begg [33] and Egger tests [34]. Meta-analyses were conducted in May 2021.

We conducted per-protocol subgroup analyses to examine adherence based on complexity of the lifestyle interventions (two lifestyle recommendations vs. more than two lifestyle recommendations); use of strategies to promoting adherence (no use vs. use); total duration of the intervention (≤34 weeks vs. >34 weeks); presence of physical comorbidity (depression and comorbid condition vs. depression alone); and depression severity (moderate vs. mild).

In addition, we calculated the weighted mean proportion of sessions completed by the participants and pooled data of the proportion of dropouts at post-treatment assessment following the same methodology described above.

## 3. Results

### 3.1. Study Selection

**Figure 1:**
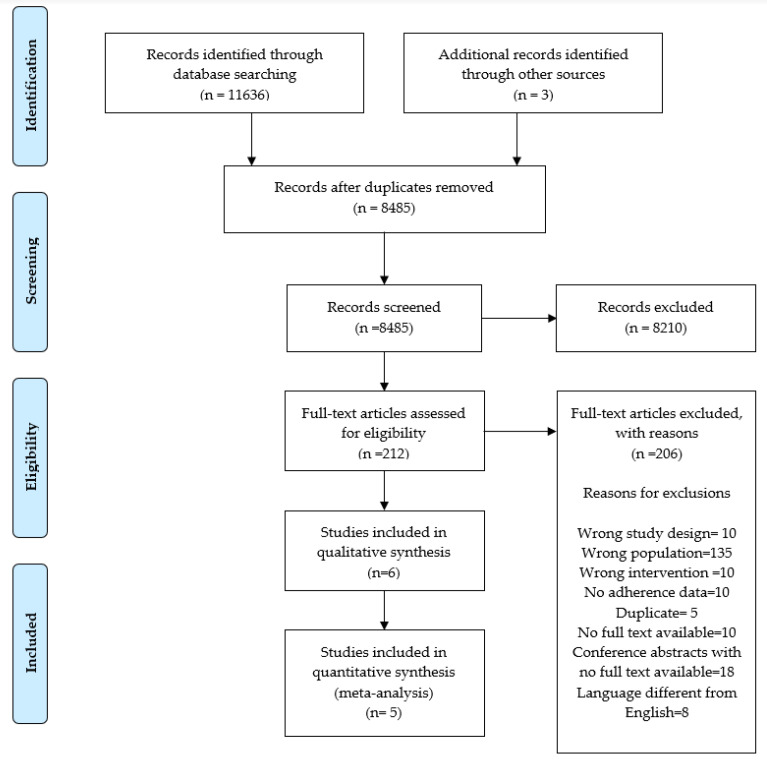
PRISMA flow diagram.

A total of 11,636 records were identified through the database searches (8485 unique records after deduplication). Of these, 209 were examined at full-text level. Three additional records identified through manual review (backward citation search) were also examined at full-text level. After the full-text evaluation of the 212 studies, we finally included six studies in the systematic review and five of them in the meta-analysis (Pagoto et al., 2013 [26] was not included in this analysis due to missing the number of completers data) (Figure 1).

### 3.2. Characteristics of the Included Studies

The characteristics of the included studies are available in Table 1. The total sample size across the included RCTs was 1138 participants. All studies were conducted in the USA except for one in Spain [35]. All participants were adults, and the majority were female (range: 70–100%). Five trials included participants with specific comorbidities such as overweight/obesity and polycystic ovary syndrome [36], overweight/obesity and type 2 diabetes [37], and obesity [26,27,38]. According to the severity of depression at baseline, two studies included patients with mild depression [27,37] and four included patients with moderate depression [26,35,36,38]. One study was conducted in a tertiary polycystic ovary syndrome centre [36], two were conducted in community clinics [26,37], one was conducted in family and internal medicine departments [27] and two were conducted in primary care centres [35,38].

Two studies compared the lifestyle intervention to usual care [27,37], one compared the lifestyle intervention to an improved treatment as usual and two active treatments [35], and the remaining studies compared the lifestyle with an active treatment [26,36,38].

Four studies focused on nutrition and exercise [26,27,36,37,38], whereas one study focused on diet, exercise, and sleep [35]. Intervention duration ranged from 4 to 52 weeks. The mean number of treatment session was “21.3” sessions (range: 5–38 sessions). Two studies included face-to-face sessions [36,37], one study included face-to-face sessions and phone calls [26], one study included face-to-face sessions, videos, and telephone sessions [27], and one study included face-to-face sessions and web-based modules [35]. Two studies used individual sessions [27,36], three studies used both individual and group sessions [26,35,37], and one used group sessions [38].

Regarding the characteristics of the lifestyle interventions, in Cooney et al., 2018 [36], participants received 16 face-to-face and individual 30-minute weekly nutrition and exercise counselling visits delivered by a counsellor. Regarding the nutrition, a self-selected diet around 1500–1800 kcal/day of foods included on the Food Guide Pyramid was recommended to the participants. Regarding the exercise, the objective recommended was starting at 50 min/week and up to 175 min/week.

In Moncrieft et al., 2016 [37], the CALM-D Intervention consisted of diet and physical activity according to the Diabetes Prevention Program protocol combined with cognitive behavioural and social learning strategies to manage depressive symptoms. At the beginning of the intervention, participants received a weight loss objective, which was 7% of initial body weight). To accomplish this aim, participants received recommendations for physical activity (practise 150 min aerobic activity/week) and caloric intake (according to the initial body weight).

In Pagoto et al., 2013 [26], The Lifestyle Intervention, based on The Diabetes Prevention Program (DPP) Lifestyle Intervention protocol [39], was delivered by a dietitian and exercise physiologist. Participants received calorie aims in order to lose 0.5–1 kg every week. They also were asked to work toward the aim of 30 min of moderate physical activity 5 days per week.

The 12-month intervention in Ma et al., 2019 [27], included components of two effective treatments for patients with both obesity and depression: The Group Lifestyle Balance (GLB) [40] program adapted from the Diabetes Prevention Program [41] and The Program to Encourage Active, Rewarding Lives for Seniors (PEARLS) [42,43]. The GLB consisted of videos for self-study for weight loss and cardiometabolic risk reduction in primary care. This program used a target-based technique to encourage weight loss by healthy dietary recommendations with reductions of 500 to 1000 kcal every day and at least 150 min of moderate-intensity physical activity every week. The PEARLS used problem-solving therapy with behavioural activation approaches as the first-line treatment and then complemented with as-needed stepwise increases in doses and number of antidepressant medications.

In Gili et al., 2020 [35], treatment consisted of one in-person and four web-based individual modules. The main aim of the in-person session was to clarify to the participants the program structure and the treatment components. The web-based contents of the psychoeducational program for the promotion of a healthy lifestyle were centred on the influence of healthy lifestyle in mental health and well-being and on the provision of structured hygienic–dietary recommendations through physical activity, diet, and sleep.

Finally, Linde et al., 2011 [38], used a behavioural weight loss intervention. Intervention session content was focused on behavioural objective setting and self-monitoring of caloric intake, physical activity, and body weight. Participants were recommended daily caloric intake objectives of 1200 or 1500 kcal to generate a loss of 1 to 2 lb every week, depending on the initial body weight. They were also asked to decrease fat intake to 20% of daily caloric intake. Physical activity objectives were increased every two weeks in increments of 500 kcal per week until the objective of 2500 kcal per week was achieved.

Two studies used strategies to increase adherence treatment: in the study by Moncrieft et al., 2016 [37], a therapist maintained a flexible approach to the session timetable, in some cases delivering individual sessions rather than group sessions to accommodate participant schedules. In Gili et al., 2020 [35], participants received two weekly automated mobile phone messages. Moreover, they received an email encouraging them to continue with the intervention if they did not access it within a week.

### 3.3. Risk of Bias

**Figure 2:**
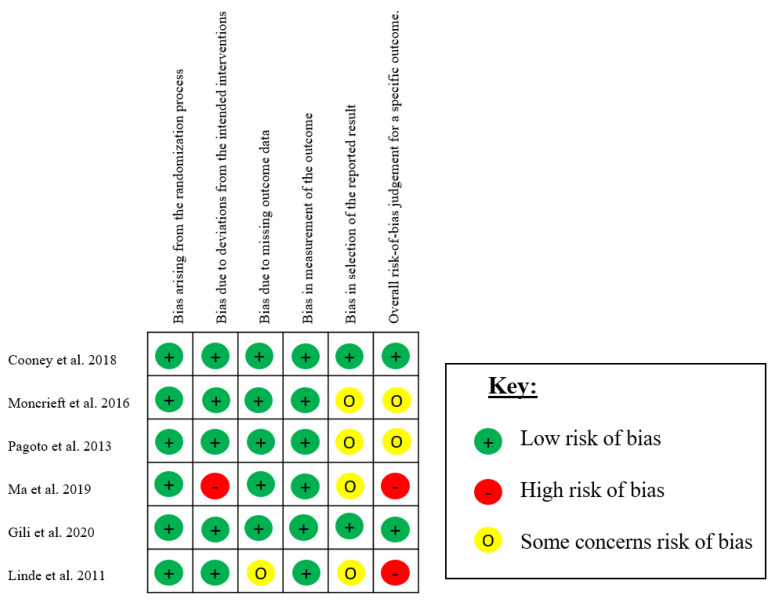
Risk of bias of included studies.

The results of the risk of bias assessment are available in Figure 2. Two studies show an overall low risk of bias [35,36], and two presented some overall concerns [26,37]. The remaining studies showed an overall high risk of bias [27,38]. All studies present low bias deriving from the randomisation process and the measurement of the outcome.

### 3.4. Adherence to the Interventions

#### 3.4.1. Meta-Analysis of Intervention Completers

**Figure 3:**
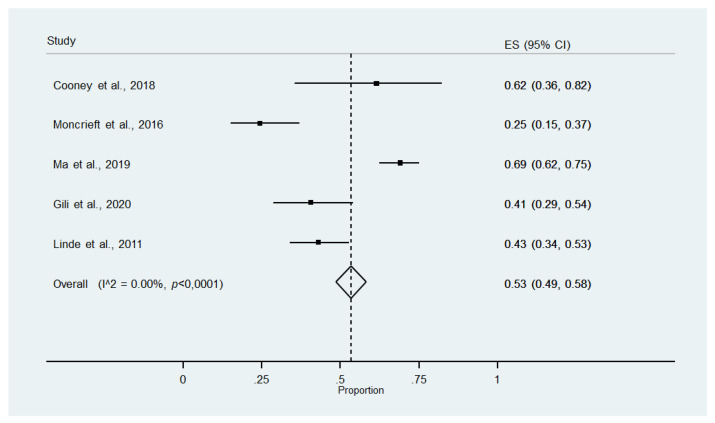
Meta-analysis of intervention completers.

Five studies provided sufficient data to determine the percentage of participants who complete the intervention (Table 2) [27,35,36,37,38]. The meta-analysis of proportions (Figure 3) showed that 53% (95% CI 49% to 58%) of the participants assigned to the intervention group completed the whole intervention.

A sensitivity analysis excluding a study [36] with shorter follow-up did not vary the results.

**Figure A1:**
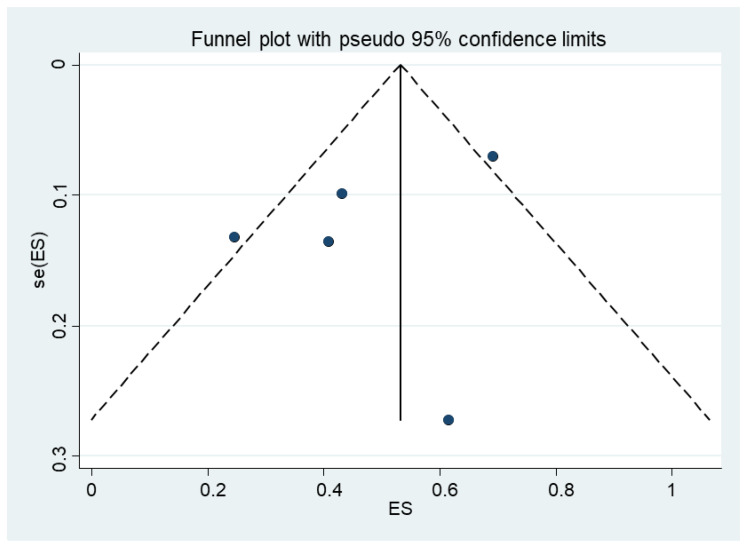
Funnel plot from the meta-analysis of intervention completers.

There was evidence of publication bias and asymmetry (Figure A1; Egger’s test: *p* = 0.040). After excluding the contributor to this bias [37], the meta-analysis of proportions showed that 58% (95% CI 53% to 63%) of participants completed the intervention.

#### 3.4.2. Mean Proportion of Sessions Completed

Five studies [26,27,35,36,38] provided sufficient data to determine the intervention adherence, which was computed in terms of mean number of sessions completed by participants divided by the total number of intervention sessions. The weighted average proportion of intervention sessions was 66% (range: 43–87%) (Table 2).

#### 3.4.3. Subgroup Analysis

There were no significant differences between groups regarding the proportion of completers in all the pre-planned subgroup analysis, except for the subgroup analysis based on the use of strategies promoting adherence (use vs. no use). Contrary to our hypothesis, the proportion of participants who adequately adhered to the interventions was higher in studies not using strategies to promote intervention adherence (61%, 95% CI 55% to 66%) than in the studies that used one or more strategies to promote intervention adherence (32%, 95% CI 24% to 41%) (Table 3).

#### 3.4.4. Meta-Analysis of Dropout Rates

**Figure 4:**
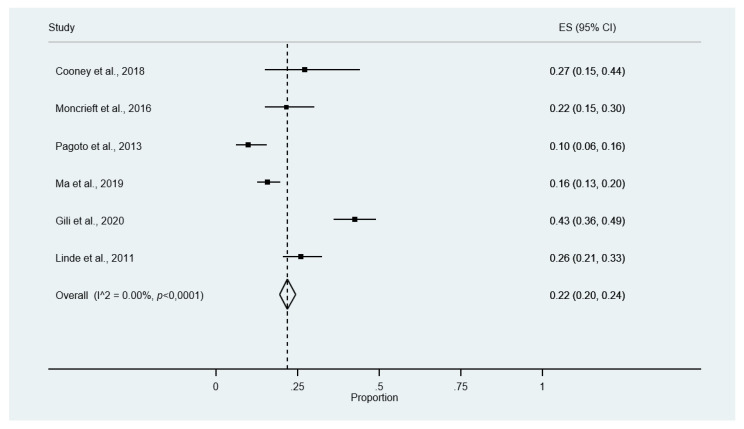
Meta-analysis of dropouts.

**Figure A2:**
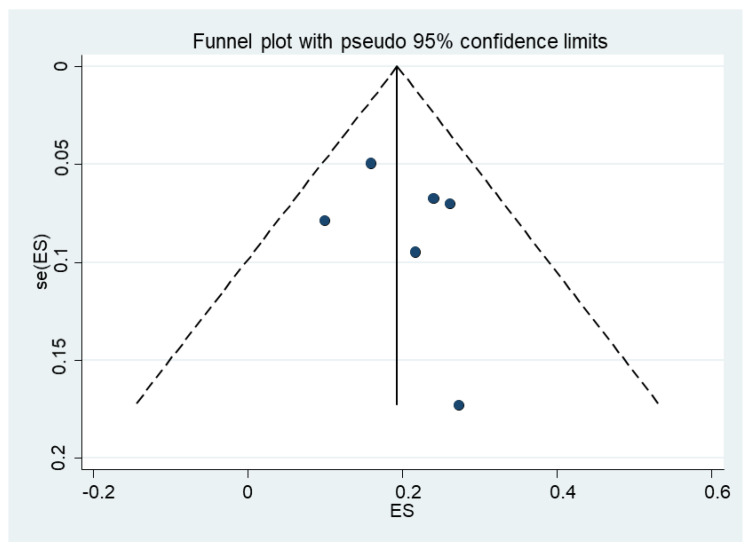
Funnel plot from the meta-analysis of dropout rates.

All studies reported the proportion of participants who failed to complete the research protocol (Table 2). The meta-analysis of proportions showed that 22% (95% CI 20% to 24%) of participants drop out of the study at post-treatment assessment (Figure 4). No evidence of publication bias and asymmetry was found in this meta-analysis (Figure A2; Egger’s test: *p* = 0.168). A sensitivity analysis excluding a study [36] with shorter follow-up did not vary the results.

## 4. Discussion

In this systematic review, we examined the adherence to lifestyle interventions for adults with depression and estimated the dropout rates in trials evaluating the impact of such interventions. We identified six trials, the majority from the USA, including 1138 participants.

The meta-analysis of intervention completers showed that around half of the participants (53%) adequately adhered to lifestyle interventions. As far as we know, this is the first meta-analysis specifically examining adherence to lifestyle interventions for adults with depression, and therefore, our results cannot be compared with those from previous systematic reviews. Nevertheless, it can be compared with other type of interventions for depression. If we compare our findings with face-to-face cognitive behaviour therapy (CBT), adherence to lifestyle interventions is substantially lower: van Ballegooijen et al., 2014 [22], carried out a meta-analysis to examine the adherence to face-to-face CBT for depression and found that 84.7% completed the entire intervention. Similar results were found by Swift and Greenberg, 2012 [44], with an adherence rate to face-to-face psychotherapy of 80%.

The adherence rate to lifestyle interventions observed in our study of people with depression is similar to the adherence rates observed in other population groups. A meta-analysis of 27 studies of weight loss interventions showed an adherence rate of 60.5% (95%CI 53.6 to 67.2) [45].

When compared with medication adherence, results are diverse. While studies of adherence to medication in depressive patients reveal around 65% of patients show good medication adherence [46], others determine that around 50% of participants discontinue antidepressant medication precipitately [47]. In any case, our results are broadly in line with those studies.

We also calculated the mean proportion of sessions completed by the participants. The reason to calculate adherence in this way is because it takes into consideration the data from all the participants, including those who do not complete the whole intervention [22]. Our observed mean proportion of completed sessions for lifestyle interventions (66%) is lower than the one observed by van Ballegooijen et al., 2014 [22], for face-to-face CBT (83%). As we observed, adherence to lifestyle interventions is lower than adherence to CBT in the two ways we measure adherence. A possible explanation for this fact could be that one can expect that, in general, acquiring a healthy lifestyle is not easy, and it can become even more difficult in people with a depressive disorder due to the symptoms.

A large number of subgroup analyses were performed to explore the extent to which certain population or intervention characteristics moderated treatment adherence. Nevertheless, statistically significant differences were only found in one subgroup analysis based on the use of strategies to promoting adherence (use vs. not use). Contrary to our expectations, adherence was higher among those studies that did not use strategies to promote intervention adherence (61%, 95%CI 55% to 66%) than in those that used some strategy to support adherence (32%, 95%CI 24% to 41%). It may be argued that those interventions that use certain strategies to promote treatment adherence, such as telephone or email contact, reminders, etc., have a positive effect on the adherence rates. In fact, regarding computerised CBT, Richards and Richardson (2012) [48] found that adherence was associated with the form of guidance: the completers percentage was 72% when the computerised CBT interventions included the guide of a therapist, while the percentage of completers was 26% in those interventions with no support. Possible reasons for this discrepancy may be due to the low number of studies included in the subgroup analysis as well as the heterogeneity in terms of the intervention (e.g., face-to-face sessions vs. individual and group sessions vs. Internet-based modules) between the studies. Another possible explanation could be that one of the two studies included in the group that use strategies to promote adherence is an Internet-based intervention [35]. Previous studies show that Internet-based interventions are associated with low adherence rates [49,50,51,52,53]. In any case, more research is needed to keep investigating other characteristics that could play a role in the treatment adherence.

A meta-analysis of dropouts was also carried out to calculate the percentage of participants who failed to complete the research protocol. Our results showed that 22% of participants dropped the study at post-treatment assessment. Our result is similar from a previous meta-analysis of 47 RCTs carried out by Wong et al., 2021 [19], which estimated an attrition rate of 21% in the intervention group and 20% in the control group.

Our systematic review and meta-analysis present several limitations. First, our meta-analysis is based on a small number of studies, some of which presented a small sample size, which could limit the accuracy of our estimations. Second, there were a clinical heterogeneity in terms of clinical population (e.g., depression and depression plus comorbidity) and intervention characteristics (e.g., sessions modality, number of sessions). Third, a meta-regression analysis was planned a priori to explore the relationship between adherence and intervention impact on depression. Nevertheless, due to the low number of studies reporting on adherence, it was ultimately not possible to perform such analyses.

Finally, our adherence definition (the proportion of participants who complete the intervention coding 100% or the author’s criteria of completers) is broad, and it may affect the robustness of the analysis. The reason for defining it like this is that there is no agreed definition of adherence, and authors tend to report it in a number of different ways. Despite these limitations, our study has several strengths. To the best of our knowledge, this is the first systematic review with a meta-analysis examining the adherence to lifestyle interventions for treatment of adults with depression. Another strength is the methodological robustness: searches were made in several databases, including grey literature, and the study selection and data extraction were carried out by two independent reviewers adhering to the Centre for Reviews and Dissemination guidelines. Moreover, it is important to note that COVID-19 Mental Disorders Collaborators [54] have estimated a relevant increase in the prevalence of major depressive disorder during the pandemic to nowadays, with an increase of around 27%. In addition, previous research has demonstrated that the COVID-19 pandemic has had an adverse impact on healthy lifestyle behaviours regarding diet, physical activity, and sleep, and consequently, a decrease in mental health and quality of life [55]. For all these reasons, our results could help design future RCTs and interventions with the aim of promoting adherence to healthy lifestyle interventions for adults with depression.

Adherence analyses have been carried out in this systematic review and meta-analysis in order to better understand the adherence treatment to lifestyle interventions for patients with depression, since the correct assessment of adherence behaviour is essential for designing effective and efficient treatments [56]. Nevertheless, the small number reporting adherence data according to this definition in the studies makes its examination complicated. Moreover, there are few studies that investigate lifestyle interventions for a diagnosed depressive disorder population or with significant depressive symptomatology. For these reasons, future research is needed to develop and evaluate interventions to support adherence to lifestyle interventions in people with depression.

## 5. Conclusions

In conclusion, our findings suggest that around half of adults with depression adhere to lifestyle interventions. Regarding previous research, this result is similar to medication adherence but lower than other types of psychotherapy, such as CBT. Furthermore, subgroup analysis reveals a significant proportion of adherence completers in those studies who did not use strategies to promote treatment adherence, contrary to earlier literature. In addition, a low dropout rate was found, similar to previous literature.

## Figures and Tables

**Table 1:** Characteristics of the included studies.

Reference	Setting and Country	Sample Characteristics	Intervention	Target	Total Study Period (Months)/Intervention Duration (Weeks)	Strategies to Promote Adherence	Control
Cooney et al., 2018	Tertiary PCOS centre; USA	Overweight/obese women with PCOS. Age (median and IR): Intervention: 32 (27–34). Comparator: 29 (25–33). Percentage of women: 100%	16 weekly in-person individual face-to-face visits. N = 13.	Nutrition and exercise	4 months/16 weeks	No	CBT and LS. N = 20
Moncrieft et al., 2016	Local community health clinics; USA	Low-income, minority patients with type 2 diabetes and overweight/obesity. Age (M[SD]): Overall 54.81 (7.36). Percentage of women: 71.2%	17 sessions (2 individual sessions + 2 weekly sessions + 4 biweekly group sessions + 9 monthly group sessions). N = 57	Diet and physical activity	12 months/52 weeks	Yes	UC: N = 54
Pagoto et al., 2013	Community clinics; USA	Obese women; Age (M[SD]): Overall 45.9 (10.8). Percentage of women: 100%	38 sessions (10 individual sessions + 16 group behavioural weight loss session; 6 group monthly sessions + 6 counselling monthly phone calls). N = 83	Nutrition and exercise	12 months/52 weeks	No	BA condition: Lifestyle intervention + behaviour therapy. N = 78
Ma et al., 2019	Family and internal medicine departments within medical centres; USA	Patients with obesity; Age (M[SD]): Overall 51 (12.1). Percentage of women: 70%	26 sessions (9 in- person individual sessions + 11 home-viewed GLB videos + 6 monthly telephone sessions) + N = 204	Weight loss and physical activity	12 months/52 weeks	No	UC: N = 205
Gili et al., 2020	Primary care; Spain	Primary care patients; Age: (M[SD]): Overall 45.3 (12.63). Percentage of women: 77.83%	1 face-to-face group sessions + 4 web-based individual and interactive therapeutic modules. N = 54	Physical activity, diet, and sleep	15 months/4–8 weeks	Yes	iTAU: N = 57 Brief intervention based on mindfulness. N = 54 Positive affect promotion program. N = 56
Linde et al., 2011	Group Health primary care clinics; USA	Women with obesity. Age (average): Overall 52. Percentage of women: 100%	26 group sessions (16 weekly sessions + 4 sessions every other week + 6 monthly sessions). N = 102	Caloric intake, physical activity, and body weight	12 months/52 weeks	No	Combined weight loss/depression intervention. N = 101

PCOS: Polycystic Ovary Syndrome; USA: United States of America; IR: Interval Range; M: Median; SD: Standard Deviation; GLB: The Group Lifestyle Balance; CBT: Cognitive Behavioural Therapy; LS: Lifestyle Modification; UC: Usual Care; BA: Behavioural Activation; iTAU: Improved Treatment As Usual.

**Table 2:** Adherence outcome data (completers and mean proportion of session completed) and dropout data.

Reference	N Intervention Completers	N Total in Intervention	Mean Number of Sessions Completers (SD)	Total Number of Intervention Sessions	Mean Proportion	N Dropouts at Post-Treatment Assessment	N Total Randomised
Cooney et al., 2018	8	13	14 (NR)	16	0.87	9	33
Moncrieft et al., 2016	14	57	NR	17	NA	24	111
Pagoto et al., 2013	NR	83	17.95 (8.59)	26	0.69	16	161
Ma et al., 2019	141	204	11.42 (5.07)	15	0.76	65	409
Gili et al., 2020	22	54	3.09 (1.91)	5	0.61	94	221
Linde et al., 2011	44	102	11.3 (8.4)	26	0.43	53	203
Total weighted average proportion					0.6616		

SD: Standard Deviation; NA: Not Applicable; NR: Not Reported.

**Table 3:** Subgroup meta-analysis of the adherence of healthy lifestyle interventions for adults with depression.

Subgroup Analysis	References	ES (95% CI)
Intervention complexity		
Two lifestyle recommendations	Cooney et al., 2018; Moncrieft et al., 2016; Ma et al., 2019; Linde et al., 2011	0.55 (0.50–0.60)
More than two lifestyle recommendations	Gili et al., 2020	0.41 (0.29–0.54)
Use of strategies to promoting adherence		
No use	Cooney et al., 2018; Ma et al., 2019; Linde et al., 2011	0.61 (0.55–066)
Use	Moncrieft et al., 2016; Gili et al., 2020	0.32 (0.24–0.41)
Total duration of the intervention		
≤34 weeks	Cooney et al., 2018; Gili et al., 2020	0.45 (0.32–0.57)
>34 weeks	Moncrieft et al., 2016; Ma et al., 2019; Linde et al., 2011	0.55 (0.50–0.60)
Characteristics of the sample		
Depression and comorbid condition	Cooney et al., 2018; Moncrieft et al., 2016; Ma et al., 2019; Linde et al., 2011	0.55 (0.50–0.60)
Depression alone	Gili et al., 2020	0.41 (0.29–0.54)
Depression severity at baseline		
Moderate	Cooney et al., 2018; Gili et al., 2020; Linde et al., 2011	0.44 (0.36–0.51)
Mild	Moncrieft et al., 2016; Ma et al., 2019	0.60 (0.53–0.65)

CI: Confidence Intervals.

## Data Availability

The data presented in this study are available on request from the corresponding author.

## Appendix A

**Table A1:** Search strategy.

**No**	**Queries (PubMed Web)**	**Results**
#1	depression[mesh]	213,567
#2	“depressive disorder”[mesh]	108,151
#3	depress*[Title/Abstract]	456,470
#4	“depressive disorder*”[Title/Abstract]	36,144
#5	“mood disorder”[Title/Abstract]	5450
#6	#1 OR #2 OR #3 OR #4 OR #5	499,450
#7	lifestyle[mesh]	92,275
#8	“healthy lifestyle”[mesh]	5827
#9	“life style*”[Title/Abstract]	12,412
#10	lifestyle*[Title/Abstract]	102,793
#11	“behaviour change*”[Title/Abstract]	5590
#12	“health promotion*”[Title/Abstract]	32,666
#13	#7 OR #8 OR #9 OR #10 OR #11 OR #12	201,582
#14	“motor activity”[mesh]	287,417
#15	“exercise”[mesh]	192,671
#16	“sedentary behavior”[mesh]	9102
#17	“physical activit*”[Title/Abstract]	111,276
#18	“motor activit*”[Title/Abstract]	16,122
#19	sedentar*[Title/Abstract]	31,494
#20	exercice*[Title/Abstract]	476
#21	fitness[Title/Abstract]	73,633
#22	“leisure activit*”[Title/Abstract]	3540
#23	sport*[Title/Abstract]	76,812
#24	#14 OR #15 OR #16 OR #17 OR #18 OR #19 OR #20 OR #21 OR #22 OR #23	483,951
#25	Diet, Mediterranean[mesh]	3339
#26	Diet, healthy[mesh]	3560
#27	“healthy food*”[Title/Abstract]	3977
#28	healthy eat*[Title/Abstract]	6967
#29	diet*[Title/Abstract]	566,152
#30	“weight reduction*”[Title/Abstract]	9414
#31	“weight loss*”[Title/Abstract]	86,458
#32	#25 OR #26 OR #27 #28 OR #29 OR #30 OR #31	643,768
#33	#24 OR #32	1,081,234
#34	#6 AND #13 AND #33	3064

## References

[1] Kessler R.C., McGonagle K.A., Zhao S., Nelson C.B., Hughes M., Eshleman S., Wittchen H.U., Kendler K.S. (1994). Lifetime and 12-month prevalence of DSM-III-R psychiatric disorders in the United States. Results from the National Comorbidity Survey. Arch. Gen. Psychiatry.

[2] Sinyor M., Rezmovitz J., Zaretsky A. (2016). Screen all for depression. BMJ.

[3] GBD 2017 Disease and Injury Incidence and Prevalence Collaborators (2018). Global, regional, and national incidence, prevalence, and years lived with disability for 354 diseases and injuries for 195 countries and territories, 1990–2017: A systematic analysis for the Global Burden of Disease Study 2017. Lancet.

[4] Katon W., Lin E.H., Kroenke K. (2007). The association of depression and anxiety with medical symptom burden in patients with chronic medical illness. Gen. Hosp. Psychiatry.

[5] Moussavi S., Chatterji S., Verdes E., Tandon A., Patel V., Ustun B. (2007). Depression, chronic diseases, and decrements in health: Results from the World Health Surveys. Lancet.

[6] Gili M., Comas A., García-García M., Monzón S., Antoni S.B., Roca M. (2010). Comorbidity between common mental disorders and chronic somatic diseases in primary care patients. Gen. Hosp. Psychiatry.

[7] Naylor C., Parsonage M., McDaid D., Knapp M., Fossey M., Galea A. Long-Term Conditions and Mental Health The Cost of Co-Morbidities. https://www.kingsfund.org.uk/sites/default/files/field/field_publication_file/long-term-conditions-mental-health-cost-comorbidities-naylor-feb12.pdf.

[8] Hidaka B.H. (2012). Depression as a disease of modernity: Explanations for increasing prevalence. J. Affect. Disord..

[9] Dingle G.A., Sharman L.S., Haslam C., Donald M., Turner C., Partanen R., Lynch J., Draper G., van Driel M.L. (2021). The effects of social group interventions for depression: Systematic review. J. Affect. Disord..

[10] Lopresti A.L., Hood S.D., Drummond P.D. (2013). A review of lifestyle factors that contribute to important pathways associated with major depression: Diet, sleep and exercise. J. Affect. Disord..

[11] Cuijpers P., van Straten A., Andersson G., van Oppen P. (2008). Psychotherapy for depression in adults: A meta-analysis of comparative outcome studies. J. Consult. Clin. Psychol..

[12] Cuijpers P., Reynolds C.F., Donker T., Li J., Andersson G., Beekman A. (2012). Personalized treatment of adult depression: Medication, psychotherapy, or both? A systematic review. Depress. Anxiety.

[13] Khan A., Faucett J., Lichtenberg P., Kirsch I., Brown W.A. (2012). A systematic review of comparative efficacy of treatments and controls for depression. PLoS ONE.

[14] Sarris J., O’Neil A., Coulson C.E., Schweitzer I., Berk M. (2014). Lifestyle medicine for depression. BMC Psychiatry.

[15] Molendijk M., Molero P., Ortuño Sánchez-Pedreño F., Van der Does W., Angel Martínez-González M. (2018). Diet quality and depression risk: A systematic review and dose-response meta-analysis of prospective studies. J. Affect. Disord..

[16] Imboden C., Gerber M., Beck J., Holsboer-Trachsler E., Pühse U., Hatzinger M. (2020). Aerobic exercise or stretching as add-on to inpatient treatment of depression: Similar antidepressant effects on depressive symptoms and larger effects on working memory for aerobic exercise alone. J. Affect. Disord..

[17] Ho F.Y., Chan C.S., Lo W.Y., Leung J.C. (2020). The effect of self-help cognitive behavioral therapy for insomnia on depressive symptoms: An updated meta-analysis of randomized controlled trials. J. Affect. Disord..

[18] Gómez-Gómez I., Bellón J.Á., Resurrección D.M., Cuijpers P., Moreno-Peral P., Rigabert A., Maderuelo-Fernández J.Á., Motrico E. (2020). Effectiveness of universal multiple-risk lifestyle interventions in reducing depressive symptoms: Systematic review and meta-analysis. Prev. Med..

[19] Wong V.W., Ho F.Y., Shi N.K., Sarris J., Chung K.F., Yeung W.F. (2021). Lifestyle medicine for depression: A meta-analysis of randomized controlled trials. J. Affect. Disord..

[20] Wang X., Arafa A., Liu K., Eshak E.S., Hu Y., Dong J.Y. (2021). Combined healthy lifestyle and depressive symptoms: A meta-analysis of observational studies. J. Affect. Disord..

[21] Young C.L., Trapani K., Dawson S., O’Neil A., Kay-Lambkin F., Berk M., Jacka F.N. (2018). Efficacy of online lifestyle interventions targeting lifestyle behaviour change in depressed populations: A systematic review. Aust. N. Z. J. Psychiatry.

[22] van Ballegooijen W., Cuijpers P., van Straten A., Karyotaki E., Andersson G., Smit J.H., Riper H. (2014). Adherence to Internet-based and face-to-face cognitive behavioural therapy for depression: A meta-analysis. PLoS ONE.

[23] Centre for Reviews and Dissemination (CRD) (2009). Systematic Reviews: Centre for Reviews and Dissemination’s Guidance for Undertaking Reviews in Health Care.

[24] Moher D., Liberati A., Tetzlaff J., Altman D.G., PRISMA Group (2009). Preferred reporting items for systematic reviews and meta-analyses: The PRISMA statement. PLoS Med..

[25] Ouzzani M., Hammady H., Fedorowicz Z., Elmagarmid A. (2016). Rayyan-a web and mobile app for systematic reviews. Syst. Rev..

[26] Pagoto S., Schneider K.L., Whited M.C., Oleski J.L., Merriam P., Appelhans B., Ma Y., Olendzki B., Waring M.E., Busch A.M. (2013). Randomized controlled trial of behavioral treatment for comorbid obesity and depression in women: The Be Active Trial. Int. J. Obes..

[27] Ma J., Rosas L.G., Lv N., Xiao L., Snowden M.B., Venditti E.M., Lewis M.A., Goldhaber-Fiebert J.D., Lavori P.W. (2019). Effect of Integrated Behavioral Weight Loss Treatment and Problem-Solving Therapy on Body Mass Index and Depressive Symptoms Among Patients with Obesity and Depression: The RAINBOW Randomized Clinical Trial. JAMA.

[28] Sterne J., Savović J., Page M.J., Elbers R.G., Blencowe N.S., Boutron I., Cates C.J., Cheng H.Y., Corbett M.S., Eldridge S.M. (2019). RoB 2: A revised tool for assessing risk of bias in randomised trials. BMJ.

[29] Nyaga V.N., Arbyn M., Aerts M. (2014). Metaprop: A Stata command to perform meta-analysis of binomial data. Arch. Public Health.

[30] Freeman M.F., Tukey J.W. (1950). Transformations related to the angular and the square root. Ann. Math. Statist..

[31] Miller J.J. (1978). The Inverse of the Freeman—Tukey Double Arcsine Transformation. Am. Stat..

[32] Deeks J.J., Higgins J.P., Altman D.G., Group CSM (2019). Analysing data and undertaking meta-analyses. Cochrane Handbook for Systematic Reviews of Interventions.

[33] Begg C.B., Mazumdar M. (1994). Operating characteristics of a rank correlation test for publication bias. Biometrics.

[34] Egger M., Smith G.D., Schneider M., Minder C. (1997). Bias in meta-analysis detected by a simple, graphical test. BMJ.

[35] Gili M., Castro A., García-Palacios A., Garcia-Campayo J., Mayoral-Cleries F., Botella C., Roca M., Barceló-Soler A., Hurtado M.M., Navarro M. (2020). Efficacy of Three Low-Intensity, Internet-Based Psychological Interventions for the Treatment of Depression in Primary Care: Randomized Controlled Trial. J. Med. Int. Res..

[36] Cooney L.G., Milman L.W., Hantsoo L., Kornfield S., Sammel M.D., Allison K.C., Epperson C.N., Dokras A. (2018). Cognitive-behavioral therapy improves weight loss and quality of life in women with polycystic ovary syndrome: A pilot randomized clinical trial. Fertil. Steril..

[37] Moncrieft A.E., Llabre M.M., McCalla J.R., Gutt M., Mendez A.J., Gellman M.D., Goldberg R.B., Schneiderman N. (2016). Effects of a Multicomponent Life-Style Intervention on Weight, Glycemic Control, Depressive Symptoms, and Renal Function in Low-Income, Minority Patients With Type 2 Diabetes: Results of the Community Approach to Lifestyle Modification for Diabetes Randomized Controlled Trial. Psychosom. Med..

[38] Linde J.A., Simon G.E., Ludman E.J., Ichikawa L.E., Operskalski B.H., Arterburn D., Rohde P., Finch E.A., Jeffery R.W. (2011). A randomized controlled trial of behavioral weight loss treatment versus combined weight loss/depression treatment among women with comorbid obesity and depression. Ann. Behav. Med..

[39] Diabetes Prevention Program (DPP) Research Group (2002). The Diabetes Prevention Program (DPP): Description of lifestyle intervention. Diabetes Care.

[40] Kramer M.K., Kriska A.M., Venditti E.M., Miller R.G., Brooks M.M., Burke L.E., Siminerio L.M., Solano F.X., Orchard T.J. (2009). Translating the Diabetes Prevention Program: A comprehensive model for prevention training and program delivery. Am. J. Prev. Med..

[41] Knowler W.C., Barrett-Connor E., Fowler S.E., Hamman R.F., Lachin J.M., Walker E.A., Nathan D.M., Diabetes Prevention Program Research Group (2002). Reduction in the incidence of type 2 diabetes with lifestyle intervention or metformin. N. Engl. J. Med..

[42] Ciechanowski P., Wagner E., Schmaling K., Schwartz S., Williams B., Diehr P., Kulzer J., Gray S., Collier C., LoGerfo J. (2004). Community-integrated home-based depression treatment in older adults: A randomized controlled trial. JAMA.

[43] Ciechanowski P., Chaytor N., Miller J., Fraser R., Russo J., Unutzer J., Gilliam F. (2010). PEARLS depression treatment for individuals with epilepsy: A randomized controlled trial. Epilepsy Behav..

[44] Swift J.K., Greenberg R.P. (2012). Premature discontinuation in adult psychotherapy: A meta-analysis. J. Consult. Clin. Psychol..

[45] Lemstra M., Bird Y., Nwankwo C., Rogers M., Moraros J. (2016). Weight loss intervention adherence and factors promoting adherence: A meta-analysis. Patient Prefer. Adherence.

[46] Roca M., Armengol S., Salvador-Carulla L., Monzón S., Salvà J., Gili M. (2011). Adherence to medication in depressive patients. J. Clin. Psychopharmacol..

[47] Sansone R.A., Sansone L.A. (2012). Antidepressant adherence: Are patients taking their medications?. Innov. Clin. Neurosci..

[48] Richards D., Richardson T. (2012). Computer-based psychological treatments for depression: A systematic review and meta-analysis. Clin. Psychol. Rev..

[49] Batterham P.J., Neil A.L., Bennett K., Griffiths K.M., Christensen H. (2008). Predictors of adherence among community users of a cognitive behavior therapy website. Patient Prefer. Adherence.

[50] Donkin L., Glozier N. (2012). Motivators and motivations to persist with online psychological interventions: A qualitative study of treatment completers. J. Med. Int. Res..

[51] Gilbody S., Littlewood E., Hewitt C., Brierley G., Tharmanathan P., Araya R., Barkham M., Bower P., Cooper C., Gask L. (2015). Computerised cognitive behaviour therapy (cCBT) as treatment for depression in primary care (REEACT trial): Large scale pragmatic randomised controlled trial. BMJ.

[52] Karyotaki E., Kleiboer A., Smit F., Turner D.T., Pastor A.M., Andersson G., Berger T., Botella C., Breton J.M., Carlbring P. (2015). Predictors of treatment dropout in self-guided web-based interventions for depression: An ‘individual patient data’ meta-analysis. Psychol. Med..

[53] Gilbody S., Brabyn S., Lovell K., Kessler D., Devlin T., Smith L., Araya R., Barkham M., Bower P., Cooper C. (2017). Telephone-supported computerised cognitive-behavioural therapy: REEACT-2 large-scale pragmatic randomised controlled trial. Br. J. Psychiatry.

[54] COVID-19 Mental Disorders Collaborators (2021). Global prevalence and burden of depressive and anxiety disorders in 204 countries and territories in 2020 due to the COVID-19 pandemic. Lancet.

[55] Caroppo E., Mazza M., Sannella A., Marano G., Avallone C., Claro A.E., Janiri D., Moccia L., Janiri L., Sani G. (2021). Will Nothing Be the Same Again?: Changes in Lifestyle during COVID-19 Pandemic and Consequences on Mental Health. Int. J. Environ. Res. Public Health.

[56] Sabaté E. (2003). Adherence to Long-Term Therapies: Evidence for Action.

